# Effects of Preventive Acupuncture and Moxibustion on Fat Accumulation, Blood Lipid, and Uterus *E*_2_ of Menopause Rats

**DOI:** 10.1155/2014/621975

**Published:** 2014-02-04

**Authors:** Shi-Peng Zhu, Yu-wei He, Huan Chen, Zhi-Fang Sun, Na Ding, Jie Mo, Bing-Yan Cao, Li Luo, Qing-Qing Zhang, Yang Wang, Lu-Fen Zhang, Xiao-Xuan Ren, Meng-Wei Guo, Ya-Fang Zhao, Liang-Xiao Ma, Xiao-Hong Li

**Affiliations:** ^1^School of Acupuncture, Moxibustion and Tuina, Beijing University of Chinese Medicine, Beijing 100029, China; ^2^Huguosi Hospital Affiliated to Beijing University of Chinese Medicine, Beijing 100035, China; ^3^Medical College of Hangzhou Normal University, Hangzhou 310000, China; ^4^Yangzhou Maternal and Child Health Hospital, Yangzhou 225000, China; ^5^Graduate School of Beijing University of Chinese Medicine, Beijing 100029, China

## Abstract

*Objective*. To observe the effect of preventive acupuncture and moxibustion on blood lipid of menopause rats. *Methods*. Seventy 10-month-old SD rats with estrous cycle disorders were divided into three control groups and four treatment groups (*n* = 10/group) and another ten 3.5-month-old female SD rats were chosen as young control group. Preventive acupuncture and moxibustion were applied at Guanyuan (CV 4). Body weight growth rate has been recorded. Plasma total cholesterol (TC), triglyceride (TG), low density lipoprotein (LDL), and high density lipoprotein (HDL) levels and uterus *E*_2_ level were measured. *Results*. Compared to young control group, plasma TC and LDL increased and uterus *E*_2_ reduced significantly in 12-month-old control group. Compared to 12-month-old control group, plasma TC and LDL level and body weight growth rate decreased while HDL level increased remarkably in preventive acupuncture 12-month-old group. Compared to 14-month-old control group, plasma TC level and body weight growth rate decreased remarkably in preventive moxibustion 14-month-old group. *Conclusions*. Preventive acupuncture and moxibustion can significantly decrease the plasma TG and LDL, increase the plasma HDL, and prevent fat accumulation. Our finding suggests that preventive acupuncture and moxibustion have beneficial effects on blood lipid. Different treatment effects were found between preventive acupuncture and preventive moxibustion.

## 1. Introduction

Dyslipidemia, characterized by alterations in the levels and composition of blood lipids, is a potential risk for cardiovascular disease (CVD), stroke, and peripheral artery disease [1, 2]. Previous studies showed that menopause women have an adverse development of plasma lipoproteins, ischemic heart disease, stroke, and diabetes and occurred significantly more often than premenopause [3–5]. Meanwhile, some researchers found that the rapid reduction of estrogen in women at the age of menopause might be one of the most important causes for dyslipidemia [6].

Aggressive lipid-lowering strategy, especially lowering low-density lipoprotein (LDL) cholesterol levels, will reduce the rates of coronary heart disease and ischemic stroke [7, 8]. The latest ESC/EAS guidelines for management of dyslipidemia further highlighted the aggressive lipid-lowering strategy in subjects with documented CVD [9]. Statins are the first-line agents for treatment of most dyslipidemias, which can prevent atherosclerosis and reduce the morbidity and mortality of CVD by modulating dyslipidemia actively, especially lowering low-density lipoprotein cholesterol (LDL-C) [10]. However, the application of statins might be restricted by the adverse effect on the liver function, especially in patients with old age and comorbidity.

Researches proved that traditional Chinese medicine (TCM) is an effective and safer alternative therapy for dyslipidemia. For example, Xuezhikang (an extract from red yeast rice) has been proved beneficial in the treatment of dyslipidemia by some systematic reviews and has been recommended in a guideline for China adult dyslipidemia prevention [11–13]. Acupuncture is another effective method to treat dyslipidemia. Several clinical trials demonstrated that acupuncture could remarkably decrease plasma TC, TG, and LDL levels [14–17].

Preventive acupuncture and moxibustion are important means for disease prevention in TCM. It refers to apply benign acupuncture or moxibustion stimulation on the body before the intrusion of diseases, in order to improve the resistance. Previous animal experiments proved that preventive acupuncture and moxibustion could adjust hormones [18], resist damages from free radicals [19], and regulate inflammatory cytokines [20] through the neuroendocrine immune network. Furthermore, it is widely accepted that acupuncture facilitates the release of certain neurotransmitters in the central nerve system and activates either sympathetic or parasympathetic nervous systems, which elicits profound psychophysical responses of immune and endocrine systems [21, 22]. Since the regulation of neuroendocrine immune network is shared mechanism of acupuncture and preventive acupuncture and moxibustion, we hypothesized that the latter may also have certain effects on dyslipidemia. In this study, we observed the effects of preventive acupuncture, and moxibustion on climacteric fat accumulation, dyslipidemia and uterus *E*_2_ in natural aging climacteric rats.

## 2. Material and Methods

### 2.1. Animals Preparation

Clean female SD rats were purchased from Beijing Vital River Laboratory Animal Technology Co. Ltd. (License number: SCXK (Beijing) 200223). Experimental animals were raised in clean cabinets with free access to food and water. A controlled environment at a temperature of (20 ± 1)°C, humidity of 50%, and 12-hour light-dark cycle was maintained throughout the study. All procedures for animal experiments were conducted in accordance with World Health Organization's International Guiding Principles for Biomedical Research Involving Animals and were approved by the Animal Care and Use Committee at Beijing University of Chinese Medicine.

### 2.2. Grouping and Treatment

#### 2.2.1. Grouping

The menopause was determined through vaginal smear method [23]. Histological changes of vaginal smears in 9.5-month-old female SD rats, stained with alkaline methylene blue solution, were observed daily under the microscope for three estrous cycles (15 d). According to cell morphology, type, and quantity, seventy 10-month-old female SD rats were screened in which estrous cycle disorders had begun to emerge (indicating the beginning of menopause), and were divided into seven groups (*n* = 10/group), namely, 10-month-old control group, 12-month-old control group, preventive acupuncture 12-month-old group, preventive moxibustion 12-month-old group, 14-month-old control group, preventive acupuncture 14-month-old group, and preventive moxibustion 14-month-old group. Besides, ten 3.5-month-old female SD rats were chosen as young control group.

#### 2.2.2. Acupuncture and Moxibustion Method

**Figure 1:**
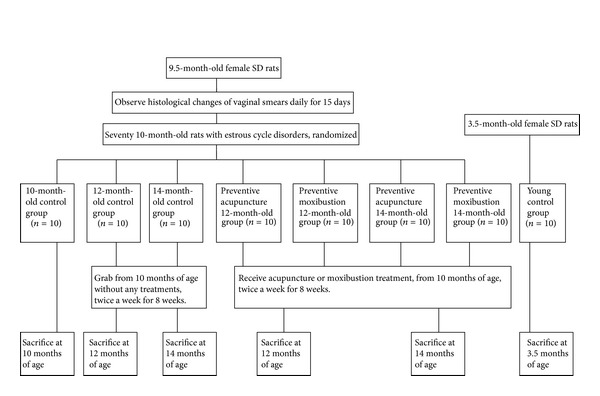
Experimental procedures.

Guanyuan (CV4) was located at the midpoint of the two hind legs roots. Acupuncture: the rats were supine on the table and fixed by the assistant's hand. The needle was directly pierced into CV4 about 0.5 cm and punctured upward. Then the needle was retained for 20 min. Moxibustion: rat hair in the region of 2 cm in diameter around CV4 was cut and the skin was exposed. The rats were supine on the table and fixed by the assistant's hand. A lit moxa cone was directly placed on the acupoint until it was burned out. Both acupuncture and moxibustion treatments were completed without anesthesia. Young control group and 10-month-old control group received no treatment. Preventive acupuncture 12-month-old group, preventive moxibustion 12-month-old group, preventive acupuncture 14-month-old group, and preventive moxibustion 14-month-old group received acupuncture or moxibustion treatment, from 10 months of age, twice a week for 8 weeks. Rats in the 12-month-old and 14-month-old control groups were only grabbed as those in the preventive acupuncture and moxibustion groups from 10 months of age without other treatments for 8 weeks (Figure 1).

### 2.3. Samples Obtaining

Materials were drawn from rats at corresponding age. Blood of rats in each group was immediately collected with rapid decapitation, placed in 2 mL anticoagulated tubes, and centrifuged (3 500 r/min, 15 min). The plasma was stored at −20°C. The above decapitated rats were placed on sterile ice. The uteri were rapidly removed, boiled in saline for 5 min, weighed, and placed in a glass homogenizer tube. One milliliter hydrogen chloride (1 mol/mL) was added. The tissues were homogenized on ice sufficiently, placed at room temperature for 100 min, and 0.8 mL sodium hydroxide (NaOH, 1 mol/mL) was added to neutralize to a certain pH. Then it was centrifuged (3 500 r/min, 15 min) and the supernatant was stored at −70°C.

### 2.4. Detection Indicators and Methods

#### 2.4.1. Measure of Body Weight Growth Rate

Body weight of all rats was measured at the 10th month and before the sacrifice. Body weight growth rate = (weight at sacrifice − 10-month-old weight)/10-month-old weight.

#### 2.4.2. Blood Lipids Assessment

These tests include plasma TC, TG, LDL, and HDL levels measurements. Biochemical methods were used in accordance with kit instructions.

#### 2.4.3. Uterus *E*_2_ Assessment

It was measured with radioimmunoassay. The process was guided strictly under instructions.

### 2.5. Statistical Analysis

Data were presented as means ± standard deviation. Differences among groups were examined using one-way ANOVA, followed by Student-Newman-Keuls test. Analyses were performed with SPSS software version 13.0; *P* < 0.05 was considered to be statistically significant.

## 3. Results

### 3.1. Effect of Preventive Acupuncture and Moxibustion on Body Weight Growth Rate

**Figure 2:**
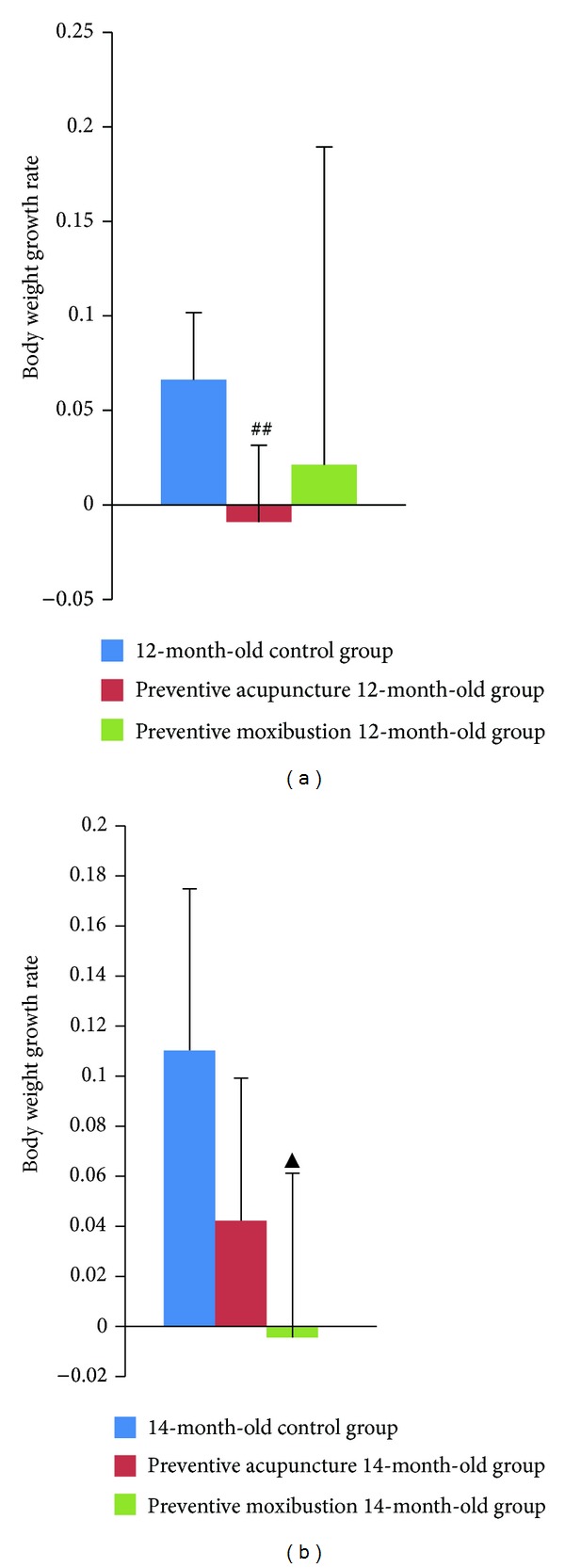
Effect of preventive acupuncture and moxibustion on body weight growth rate. Note: ^##^ *P* < 0.01, versus 12-month-old control group; ^▲^ *P* < 0.05, versus 14-month-old control group.

Preventive acupuncture 12-month-old group showed a remarkable decrease in body weight growth rate as compared to 12-month-old control group (*P* = 0.005, Figure 2(a)); body weight growth rate in preventive moxibustion 14-month-old group decreased significantly as compared to 14-month-old control group (*P* = 0.013, Figure 2(b)).

### 3.2. Effect of Preventive Acupuncture and Moxibustion on Blood Lipid

#### 3.2.1. Plasma TC Levels

**Figure 3:**
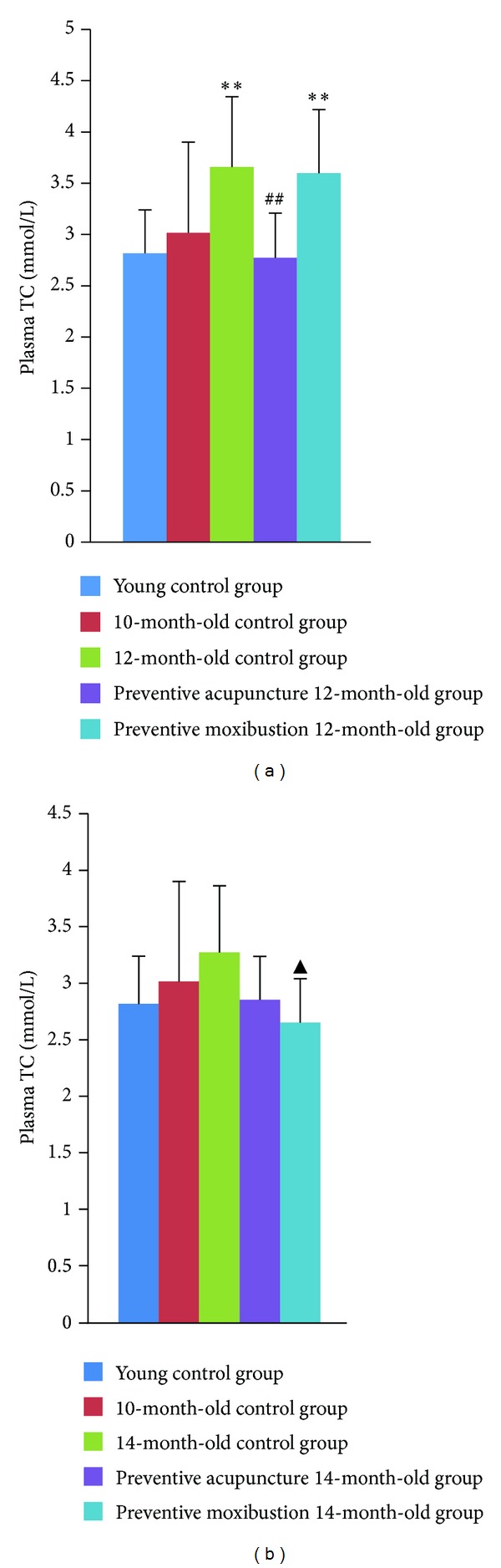
Effect of preventive acupuncture and moxibustion on plasma TC level. Note: **P < 0.01, versus young control group; ^##^ *P* < 0.01, versus 12-month-old control group; ^▲^ *P* < 0.05, versus 14-month-old control group.

Compared to young control group, plasma TC level increased significantly in 12-month-old control group (*P* = 0.002) and preventive moxibustion 12-month-old group (*P* = 0.003). Preventive acupuncture 12-month-old group showed a remarkable decrease as compared to 12-month-old control group (*P* = 0.001, Figure 3(a)). Compared to young control group, plasma TC level slightly increased in 14-month-old control group. Plasma TC level remarkably decreased in preventive moxibustion 14-month-old group as compared to 14-month-old control group (*P* = 0.018, Figure 3(b)).

#### 3.2.2. Plasma TG Levels

**Figure 4:**
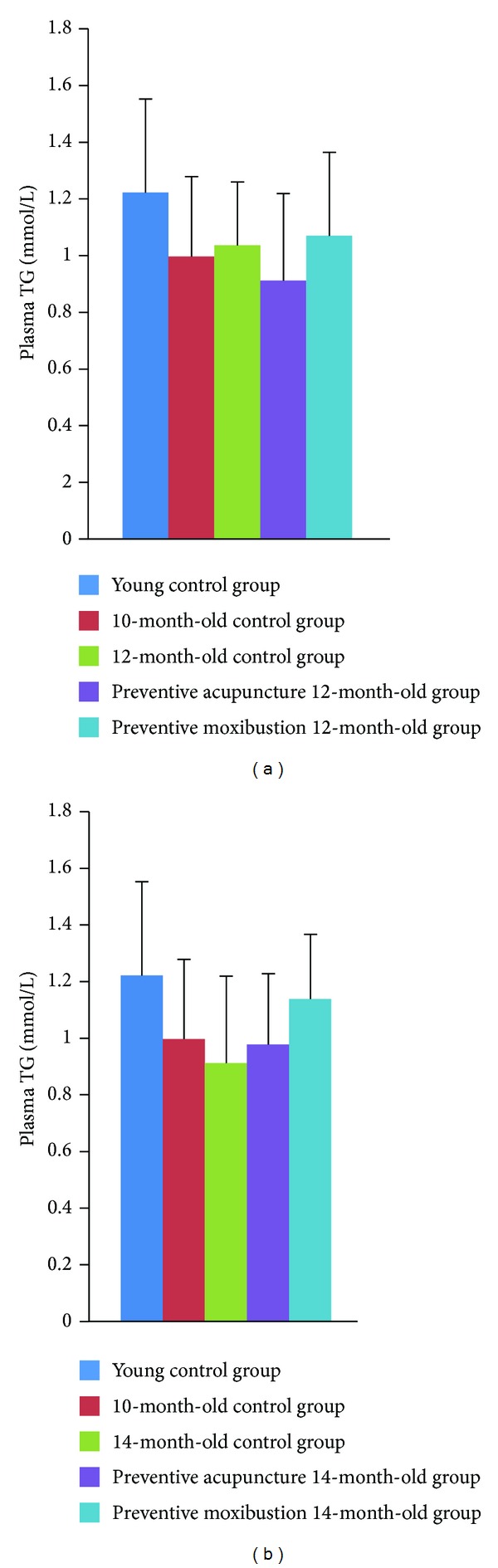
Effect of preventive acupuncture and moxibustion on plasma TG level.

No significant difference was found among all the groups (Figures 4(a) and 4(b)).

#### 3.2.3. Plasma HDL Levels

**Figure 5:**
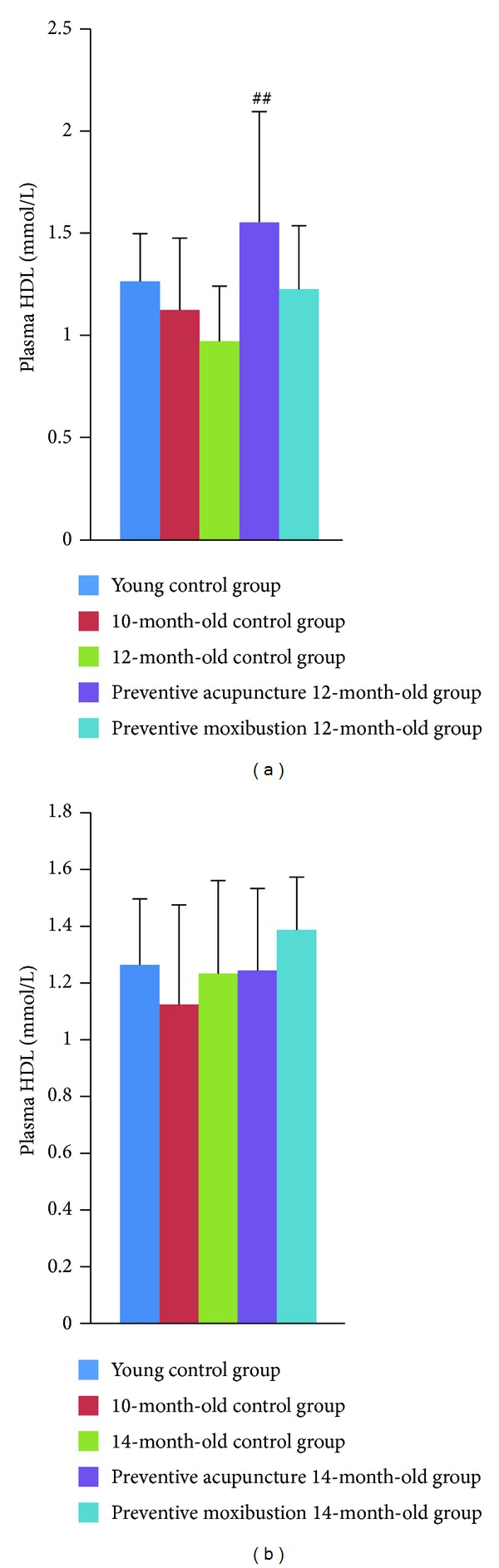
Effect of preventive acupuncture and moxibustion on plasma HDL level. Note: ^##^ *P* < 0.01, versus 12-month-old control group.

Compared to young control group, plasma HDL level showed a decreased trend in 12-month-old control group. Preventive acupuncture 12-month-old group showed a remarkable increase of HDL level as compared to 12-month-old control group (*P* < 0.001, Figure 5(a)). No significant difference was found among young control group, 14-month-old control group, preventive acupuncture 14-month-old group, and preventive moxibustion 14-month-old group (Figure 5(b)).

#### 3.2.4. Plasma LDL Levels

**Figure 6:**
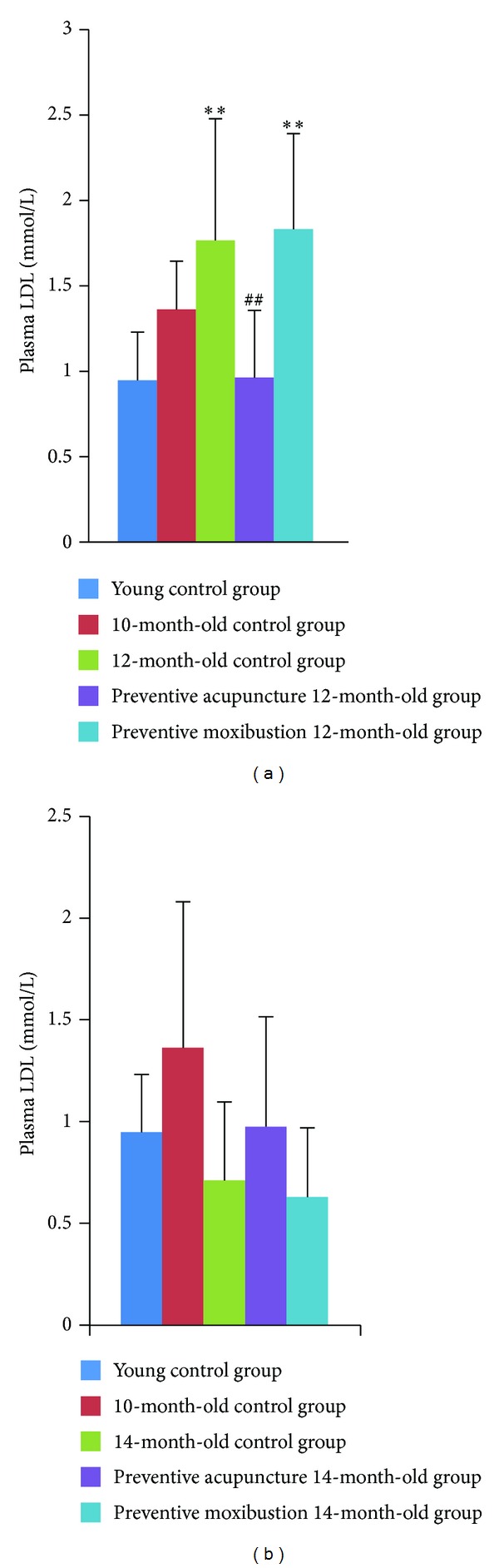
Effect of preventive acupuncture and moxibustion on plasma LDL level. Note: **P < 0.01, versus young control group; ^##^ *P* < 0.01, versus 12-month-old control group.

Plasma LDL level increased remarkably in 12-month-old control group (*P* = 0.001) and preventive moxibustion 12-month-old group (*P* < 0.001) as compared to young control group; LDL in preventive acupuncture 12-month-old group showed a significant decrease as compared to 12-month-old control group (*P* = 0.001, Figure 6(a)). No significant differences existed among young control group, 14-month-old control group, preventive acupuncture 14-month-old group, and preventive moxibustion 14-month-old group (Figure 6(b)).

### 3.3. Effect of Preventive Acupuncture and Moxibustion on Uterus *E*_2_ Level

**Figure 7:**
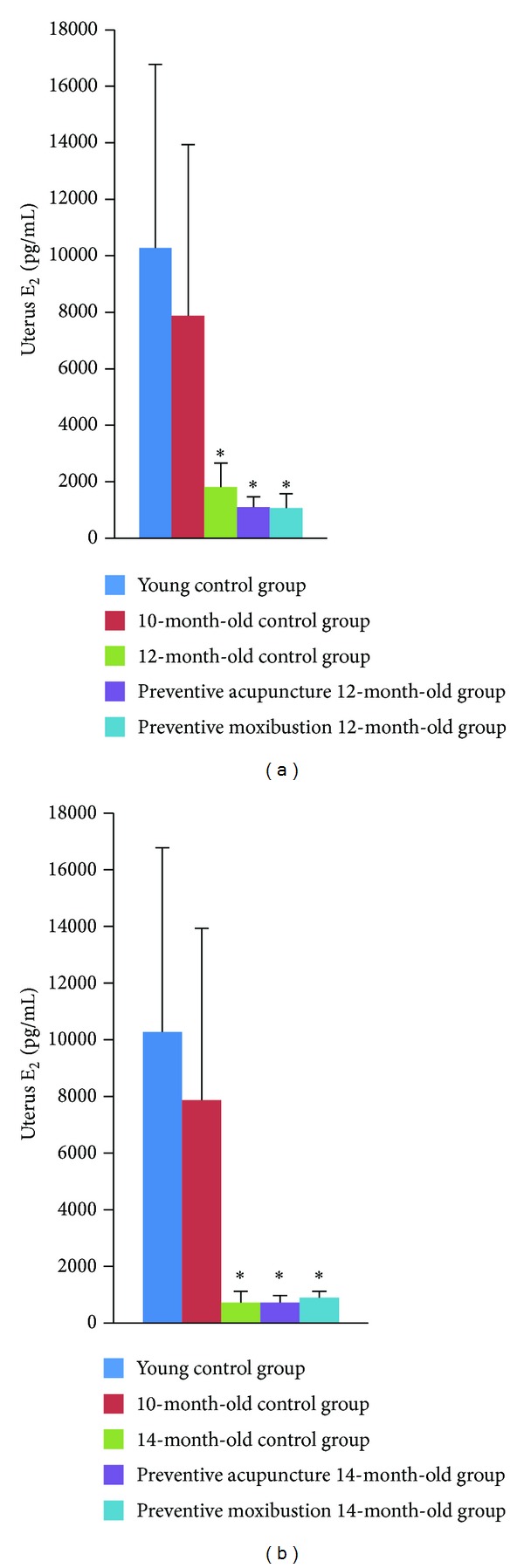
Effect of preventive acupuncture and moxibustion on uterus *E*_2_ level. Note: *P < 0.05, versus young control group.

Compared to young control group, uterus *E*_2_ level decreased significantly in 12-month-old control group (*P* = 0.049), preventive acupuncture 12-month-old group (*P* = 0.03), preventive moxibustion 12-month-old group (*P* = 0.03), 14-month-old control group (*P* = 0.024), preventive acupuncture 14-month-old group (*P* = 0.024), and preventive moxibustion 14-month-old group (*P* = 0.027, Figures 7(a) and 7(b)). No significant differences were found among 12-month-old control group, preventive acupuncture 12-month-old group, and preventive moxibustion 12-month-old group (Figure 7(a)). No significant differences were found among 14-month-old control group, preventive acupuncture 14-month-old group, and preventive moxibustion 14-month-old group (Figure 7(b)).

## 4. Discussion

Preventive acupuncture and moxibustion refer to applying acupuncture and moxibustion treatment before the occurrence of diseases. It can stimulate meridian qi and reinforce body resistance. CV4, as an important point of the Conception Vessel, has invigorating effects on the immune system and can regulate the function of the genitourinary system according to TCM theory. In this study, we observed the effects of preventive acupuncture and moxibustion at CV4 on climacteric rats' fat accumulation, lipid metabolism disorder, and the content of uterus estrogen. The major finding of the present study is that preventive acupuncture and moxibustion at CV4 can prevent fat accumulation in natural aging climacteric rats, significantly decrease the concentrations of TC and LDL, and increase the concentration of HDL in plasma. Our results suggested that preventive acupuncture and moxibustion have beneficial influence on dyslipidemia in menopause period.

Menopause is a phase of life in women that signifies the end of their reproductive period. In this period, the kidney qi gradually declines and the internal environment of body changes, which result in multisystem dysfunction. Metabolic syndrome, manifested as dyslipidemia, hypertension, and central obesity, often occurred in menopause women. It is proved that menopause increased total body weight with central obesity as well as visceral (VAT) and subcutaneous abdominal fat (SAT) [24, 25]. In our study, it was found that, compared with rats in 10-month-old control group, natural aging climacteric rats tend to have increased body weight at the 12th and 14th months, which indicates fat accumulation during climacteric period. We also found that both preventive acupuncture and preventive moxibustion could prevent excessive weight gaining in climacteric rats. It is worthwhile to note that preventive acupuncture and preventive moxibustion have differential effects on body weight growth rate. Preventive acupuncture has a more rapid effect, which can be observed in the 12th month, while preventive moxibustion has cumulative effects that are more obvious in the 14th month. The reason may be that acupuncture and moxibustion are different forms of stimuli that could trigger different regulatory pathways from the central to the peripheral and finally lead to various therapeutic effects [26, 27].

Dyslipidemia is another important feature of menopausal metabolic syndrome. Several studies reported that women who had recently entered the menopause get increased concentrations of TC, TG, and LDL [4, 28]. Others demonstrated that menopausal women have comparatively lower HDL cholesterol levels compared with premenopausal women [29]. Results of this study showed that rats' blood lipid, especially plasma TC and LDL, had an adverse change along with the time. They started to increase at 10th month and increased significantly at the 12th month compared to young control group. At the 14th month, TC was still higher than that of the young control group, while LDL tended to decrease. Moreover, plasma HDL level decreased at the 12th month. Plasma TG showed no drastic changes along with the time. Therefore, we believe that natural aging climacteric rats experienced dyslipidemia. Clinical trials have proved that acupuncture has positive effects on blood lipid since it could decrease the levels of TC, TG, and LDL [14–16]. However, there are different results for acupuncture and moxibustion's effects on HDL. Some researchers found out that acupuncture and moxibustion can significantly increase HDL-C [15, 16] while others reported no significant effects [17]. In our study, we found that, compared to 12-month-old control group, rats in preventive acupuncture 12-month-old group were of significantly decreased plasma TG and LDL and increased HDL concentrations. This indicates that preventive acupuncture has a beneficial effect on climacteric dyslipidemia. However, the effect of preventive moxibustion is not found at the 12th month. Compared with 14-month-old group, both preventive acupuncture and preventive moxibustion reduced plasma TC concentration and the latter has more obvious effect. The differential effects of preventive acupuncture and moxibustion on dyslipidemia indicate that preventive acupuncture leads to a more quick reaction, while preventive moxibustion takes cumulative effect. Moreover, the data in this study shows that both preventive acupuncture and preventive moxibustion have no significant effect on plasma TG which is inconsistent with some previous researches [16, 17]. This may be explained by the time point of intervention (in our study we begin to intervene before the onset of the disease) and the application of different acupoints.

Although there are many reasons that contribute to menopausal metabolic syndrome, studies showed that signs and symptoms in menopausal transition are likely to be triggered by a progressive decrease of estrogenic secretion [30, 31]. Our research found that, compared with rats in the young control group, 12-month-old and 14-month-old rats had significantly decreased uterus estrogen (*E*_2_). Our result demonstrated that climacteric females are of rapidly decreased *E*_2_. We also found that preventive acupuncture and preventive moxibustion have a slight effect on uterus *E*_2_ content. Compared to 12-month-old control group, uterus *E*_2_ of preventive acupuncture 12-month-old rats and preventive moxibustion 12-month-old rats was even lower. However, uterus *E*_2_ of preventive moxibustion 14-month-old rats tends to be increased compared with that of the 14-month-old control group. We speculate that 12-month-old rats have entered menopause period and are of internal milieu disorder. Although the preventive acupuncture and moxibustion are considered as a kind of beneficial prestimulations, they may further increase the burden of the organs leading to the decrease of uterus *E*_2_. Since preventive moxibustion has cumulative effects, uterus *E*_2_ in 14-month-old rats with preventive moxibustion tended to increase. The potential mechanism remains to be determined.

## 5. Conclusion

Preventive acupuncture and moxibustion have certain preventive effects on fat accumulation and dyslipidemia during climacteric period in rats. They can significantly decrease the concentrations of plasma TG and LDL and increase the plasma HDL. There are differences between the effects of preventive acupuncture and preventive moxibustion. The former has comparatively shorter time to achieve the effect, while the latter shows a cumulative effect.
